# Efficacy of a fixed combination of permethrin 54.5% and fipronil 6.1% (Effitix®) in dogs experimentally infested with *Ixodes ricinus*

**DOI:** 10.1186/s13071-015-0805-6

**Published:** 2015-04-03

**Authors:** Stéphane Bonneau, Nadège Reymond, Sandeep Gupta, Christelle Navarro

**Affiliations:** Virbac, 13ième rue - LID, 06511 Carros, France; Nadege Savelli E.I.R.L. - 2, rue de l’église, 06 270 Villeneuve Loubet, France; 41 Milltown Gate, Blessington, County Wicklow Ireland

**Keywords:** Fipronil, Permethrin, Fixed combination, Spot-on, Tick control, *Ixodes ricinus*, Ectoparasites

## Abstract

**Background:**

Ticks are the most important vectors of disease-causing pathogens in domestic animals and are considered to be second worldwide to mosquitoes as vectors of human diseases. In Europe, *Ixodes ricinus*, the sheep tick, plays an important role as companion animal parasite but is also the primary vector of medically important diseases such as tick-borne encephalitis and Lyme borreliosis.

The present study was designed to evaluate the efficacy under laboratory conditions of a new fixed spot-on combination of fipronil and permethrin (Effitix®, Virbac) in treating and preventing tick infestations of *Ixodes ricinus* in dogs.

**Methods:**

Twelve dogs were included in this randomized, controlled, blinded laboratory study. They were randomly allocated to two groups of six dogs each according to their pre-treatment live attached *Ixodes ricinus* tick count. On day 0, the dogs from Group 2 were treated with the recommended dose of Effitix®, the dogs from Group 1 remained untreated. On days −2, 7, 14, 21, 28 and 35, all dogs were infested with 50 (±4) viable unfed adult *Ixodes ricinus* (20 ± 2 males, 30 ± 2 females). Ticks were removed and counted at 48 ± 2 hours post product administration or tick infestations.

**Results:**

Through the study, the tick attachment rates for the untreated group were greater than 25% demonstrating that adequate levels of infestation were reached on the control dogs. Based on both arithmetic and geometric means (AM and GM), Effitix® was deemed to be effective against *Ixodes ricinus* on days 2, 9, 16, 23, 30 and 37 with a percentage of efficacy of 98%, 100%, 100%, 100%, 93% and 95% respectively (AM). No clinical abnormalities were detected during the study.

**Conclusions:**

The study has shown under laboratory conditions, that Effitix® is a safe and an effective combination to treat and protect dogs from *Ixodes ricinus* up to 37 days after administration. The high immediate efficacy of 98% evaluated at 48 hours post-treatment was particularly interesting, meaning that Effitix has a curative effect against ticks (*Ixodes ricinus*) and provides a rapid control of existing *Ixodes ricinus* infestation on a dog at the time of treatment.

## Background

Ticks are among the most common and important ectoparasites on dogs worldwide [1]. Tick bites can directly harm the animal by causing mechanical irritation and inflammation of the skin but more importantly through transmission of a large range of pathogens including viruses, bacteria, protozoa and helminths that may cause severe diseases both in humans and animals. Ticks are considered to be the most important vectors of disease-causing pathogens in domestic and wild animals and second worldwide to mosquitoes as vectors of human diseases [1].

In Europe, *Ixodes ricinus* (Acari: Ixodidae) known as the sheep tick or the castor bean tick, is the primary vector of medically important disease agents like the tick-borne encephalitis complex of viruses, the *Borrelia burgdorferi* sensu lato complex, the causative agents of Lyme borreliosis, *Rickettsia*, *Babesia*, and *Anaplasma* species [2]. *I. ricinus* is by far the most common tick species recently identified in many countries and/or European regions including Scandinavia, British Isles, central Europe, France, Spain, Italy, the Balkans, and eastern Europe [3].

As an ectotherm that spends the majority of its life cycle free-living, *I. ricinus* is sensitive to climatic conditions requiring a high relative humidity (at least 80%) to survive during its off-host periods and is usually found in areas of moderate to high rainfall with vegetation that retains high humidity [3]. Typical habitats of *I. ricinus* include deciduous and coniferous woodland, heathland, moorland, rough pasture, forest and urban parks [3]. However, a recent expansion in the geographic distribution of *I. ricinus* has been described to higher latitudes and altitudes together with an increase of its abundance in Europe. The driving forces for these changes have not been totally identified but a change in climate may play an important role in certain geographic regions. In addition, other ecological changes such as how we manage habitats, the distribution and abundance of tick hosts and/or the increasing initiatives to create natural environments and the trend towards spending more time in nature for recreational activities are also important considerations, leading to an increased risk of disease caused by these pathogens, in particular of Lyme borreliosis [3-5].

It is now accepted within the scientific community and the public health authorities that veterinarians, physicians and pet owners must take measures to protect companion animals from possible ectoparasitic infestations. This protection is required not only to relieve pets from the mechanical irritation and inflammation caused by the ectoparasites but also to protect them from the potential vector-borne diseases they may carry and more importantly, to limit the risks associated with zoonotic transmission of diseases. Ectoparasite management in dogs is therefore fundamental and a perfect example of the ‘One health’ approach which aims at fostering an environment which supports healthy animals and healthy people [6-8].

Effitix® is a combination of two active ingredients: fipronil and permethrin. The use of these two active product ingredients as pesticides is well-established in agriculture, the domestic environment and veterinary medicine. Fipronil belongs to the phenylpyrazole family and has acaricidal and insecticidal properties. Fipronil’s putative mode of insecticidal action is interference with the passage of chloride ions through the gamma- aminobutyric acid (GABA)-regulated chloride ion channel, which results in uncontrolled central nervous system activity and subsequent death of the insect [9]. Permethrin belongs to the family of synthetic chemicals called pyrethroids and has well-known strong repellent effects, in particular against diptera, that are sufficient to disorientate and irritate them resulting in the absence or reduction of blood feeding (anti-feeding effect) [10]. Permethrin can also act as an acaricide and insecticide, with repellent efficacy. The spectrum of activity of permethrin includes flies, mosquitoes, fleas, ticks, lice and mites. Permethrin binds to Na^+^ channels causing a slowing of their rate of closure resulting in repetitive firing/stimulation of nerves, depolarisation and nerve block. The type I pyrethroids produce a distinct poisoning syndrome characterised by progressive fine whole body tremors, exaggerated startle response, uncoordinated muscle twitching and hyperexcitability, resulting in death of the parasite. The effects are generated largely by their action on the central nervous system.

The objectives of the study performed were to test the acaricidal effect of the combination against ticks (*I. ricinus*) in dogs.

## Methods

### Study design

This was a randomized, controlled, blinded laboratory study. The design followed the recommendations of the European guidelines for the testing and evaluation of the efficacy of antiparasitic substances for the treatment and prevention of tick and flea infestation in dogs and cats [11] and the latest World Association for the Advancement of Veterinary Parasitology guidelines to conduct laboratory studies to assess the efficacy of ectoparasiticides applied to dogs and cats [12]. Eighteen dogs (male and female) were acclimatised for the study. During the acclimatisation period, animals were assessed to determine their ability to retain ticks post-infestation. Twelve dogs that met the inclusion criteria were then included in the study and randomized according to their pre-inclusion tick count into two groups of six dogs each.

Animals assigned to Group 2 were administered Effitix®, on day 0. Animals in both groups were infested with 50 (±4) viable unfed adult *I. ricinus* (20 ± 2 males, 30 ± 2 females) on days −7 (7 days prior to administration of Effitix®), −2, 7, 14, 21, 28 and 35. On days −5 (5 days prior to administration of Effitix®), 2, 9, 16, 23, 30 and 37 (approximately 48 ± 2 h post-infestation, except for day 2 which was approximately 48 ± 2 h after the application of Effitix®), all ticks were counted and removed and categorized as described in Table 1. Efficacy was defined by the group mean live and dead engorged ticks in Group 2 when compared to the mean live tick count in the untreated control group at all time points post-treatment. On day 2, dead engorged attached ticks were not included in the analysis. An effective dose was expected to provide more than 90% reduction in tick counts compared to the control group when using arithmetic means (AM).

**Table 1:** **Categorization of ticks for counting (adapted from EMEA/CVMP/EWP/005/2000-Rev.2, 2007)**

**Category**	**General findings**	**Attachment status**
1	Live	Unengorged*
2	Live	Engorged**
3	Killed	Unengorged*
4	Killed	Engorged**

*no filling of the alloscutum evident.

**obvious or conspicuous filling of the alloscutum evident.

This study was carried out according to Good Clinical Practice [13]. The protocol was approved by the ethics committee of Charles River Laboratories Preclinical Services Ireland Ltd (reference F004\12-002).

### Selection of the dogs

Twelve healthy Beagle dogs, 5 male and 7 female, weighing 8.2 to 14.0 kg and identified by electronic transponders with unique alphanumeric codes were selected for inclusion in the study. Dogs were individually housed to prevent cross contamination. On day −7, approximately 50 (±4) viable unfed adult *I. ricinus* of mixed sex ratio (20 ± 2 males, 30 ± 2 females) were applied to clinically healthy adult dogs. On day −5 (48 ± 2 h post-infestation), the number of live attached ticks were counted and recorded and all ticks were removed. To be included in the study, at least 25% of the ticks placed on the dog on day −7 (and counted on day −5) had to be recovered as live attached.

### Treatment

Dogs in group 1 remained untreated whereas dogs in group 2 received one pipette of Effitix® [the combination (fipronil 6.1% w/vol and permethrin 54.5% w/vol)] once on day 0. Dogs were weighed on day-7 for randomization purpose and on days −1 for treatment dose adjustment. Dogs weighing between 4.1 and 10 kg received one 1.1 mL pipette and dogs weighing between 10.1 and 20 kg received one 2.2 mL pipette. The product was applied topically as a spot-on, in approximately equal volumes at two application sites: one application between the shoulder blades and the second application at the lumbar area. After parting the hair, the product was applied directly to the skin. In order to maintain blinding of the study, the animals were treated by an individual who was not involved in post-treatment assessments and observations. Dogs were observed throughout the study for any clinical abnormalities.

### Tick infestations

Dogs were infested with *I. ricinus* of European origin (Germany, Bratislava, Ireland) and genetically enriched each year with new genetic seed stock from European countries. At each infestation time point, days −2, 7, 14, 21, 28 and 35, approximately 50 viable unfed adult *I. ricinus* (20 ± 2 males, 30 ± 2 females) were applied to each dog. Each dog was infested with ticks in its housing location. The ticks were applied gently to the mid-thoracic region and allowed to crawl into the hair coat and select an attachment site. Tick infestation was performed on sedated dogs. The dogs were sedated using xylazine (20 mg/mL) (Xylapan®, Vetoquinol, Buckingham, UK or Chanazine®, Chanelle, Berkshire, IE) and ketamine (100 mg/mL) (Vetalar®, Zoetis, London, UK or Narketan 10®, Vetoquinol, Buckingham, UK) at a rate of 0.1 mL/kg each.

### Tick counts

On days 2, 9, 16, 23, 30 and 37, 48 h (±2 h) post-dosing or post-infestation, the ticks were counted and removed. The examiner(s) systematically examined the head, all dorsal and ventral areas and the legs of the dog. At each tick count and collection, the numbers of female live or killed attached ticks, and female live and/or killed free ticks were quantified and all ticks were removed from the dog. Following removal, live attached ticks and killed attached ticks from each dog were categorized on the same day as described in Table 1. The engorgement status was then determined for each tick by squashing to ensure that digested blood was present.

### Efficacy assessment

#### Tick attachment rates

For tick counts, at each time point, the number of “attached live” ticks and the “attached live” tick rates were calculated and reported for Group 1 (untreated control) to confirm the vigor of the ticks. Twenty five to fifty percent of these ticks should attach to the animal at each time point following infestation in the control group. As 30 ± 2 female ticks were applied (*I. ricinus* males do not attach to their hosts), calculations were based on 30 ticks only.

#### Acaricidal efficacy

The AM and geometric mean (GM) tick counts were calculated for each group on each evaluation day. The percentages of efficacy against ticks were calculated as follows:

$$ Efficacy\ \left(\%\right)\  against\  ticks = 100\ x\ \left(Mc\ \hbox{--}\ Mt\right)\ /\ Mc, $$

where:

Mc = AM or GM number of live ticks on dogs in the negative control group at a specific time point,

Mt = AM or GM number of live ticks and dead engorged ticks on dogs in the treated group at a specific time point.

Note: on day 2, dead engorged attached ticks were not included in the analysis as the product was applied 48 h after infestation and female ticks had time to start blood feeding [12].

### Statistical analysis

The groups were compared using one-way ANOVA with a treatment effect for days 2 and 30. For days 9, 16, 23 and 37, the comparisons were made using the Kruskal-Wallis test. The counts on these study days were not suitable for analysis using ANOVA due to violations of one or both of the normality or equal variance assumptions. The statistical unit was the individual animal. All analyses were performed using programs in SAS® (Version 9.2). All statistical tests were two-tailed with a level of significance of 5%.

## Results

All animals used in the study met the inclusion criteria and were not prohibited from inclusion by the exclusion criteria. Groups were homogeneous at baseline according to sex, age and weight (p > 0.05). All animals assigned to group 2 were successfully treated with Effitix®; all infestations, tick counts, clinical assessments and bodyweight recordings were performed at the required time-points. No clinical abnormalities were observed in any dogs during the study and no concomitant treatment had to be administered.

### Tick attachment rates

The results (Table 2) show that the experimental infestations with *I. ricinus* were successful, with retention rates on the control untreated dogs ranging between 55% (day 16) and 71% (day 2) providing a severe challenge to assess the acaricidal effect of the combination product.

**Table 2:** **Mean number of live attached ticks and attachment rate (%) in the untreated control group by study day**

	**Day 2**	**Day 9**	**Day 16**	**Day 23**	**Day 30**	**Day 37**
**Mean**	21.3 (71%)	17.0 (57%)	16.5 (55%)	19.7 (66%)	20.8 (69%)	20.7 (69%)
**Minimum**	17 (57%)	13 (43%)	12 (40%)	16 (53%)	15 (50%)	19 (63%)
**Maximum**	26 (87%)	21 (70%)	22 (73%)	25 (83%)	25 (83%)	23 (77%)
**SD**	3.1 (10%)	3.7 (12%)	3.8 (13%)	3.1 (10%)	3.8 (13%)	1.6 (5%)

Mean: Arithmetic mean, SD: standard deviation.

### Efficacy

The AM and GM number of ticks that were present on untreated control dogs and on treated animals at 48 h after treatment or after each infestation are shown in Table 3. The calculated percent efficacy (GM and AM) for each time point are given in Table 4.

**Table 3:** **Arithmetic mean and geometric mean of** ***Ixodes ricinus*** **(attached and not attached) counts 48 h after treatment (Day 2) or 48 h after each re-infestation (Days 9, 16, 23, 30, 37)**

**Day**	**Group 1: negative control**			**Group 2: treated (Effitix®)**		
	**Arithmetic mean**	**Standard deviation**	**Geometric mean**	**Arithmetic mean**	**Standard deviation**	**Geometric mean**
**+2**	21.3	3.1	21.1	0.5 ^**(1)**^	0.8	0.4
**+9**	17.5	3.4	17.2	0.0 ^**(1)**^	0.0	0.0
**+16**	16.5	3.8	16.2	0.0 ^**(1)**^	0.0	0.0
**+23**	19.7	3.1	19.5	0.0 ^**(1)**^	0.0	0.0
**+30**	20.8	3.8	20.5	1.5 ^**(1)**^	2.8	0.7
**+37**	21.2	2.1	21.1	1.0 ^**(1)**^	0.9	0.8

^1^: Group 2 differed statistically significantly (p < 0.05) from the untreated control Group 1 on all assessment days.

**Table 4:** **Percentage efficacy of Effitix® against** ***Ixodes ricinus*** **48h after treatment/infestation (geometric and arithmetic mean)**

**Means**	**Days of treatment**					
	**2** ^1^	**9** ^2^	**16** ^2^	**23** ^2^	**30** ^2^	**37** ^2^
**Arithmetic**	98 %	100 %	100 %	100 %	93 %	95 %
**Geometric**	98 %	100 %	100 %	100 %	97 %	96 %

^1:^ therapeutic efficacy of the combination against day-2 infestation.

^2^: prophylactic efficacy against weekly tick re-infestation.

For both AM and GM, immediate efficacy, based on tick reduction was equal to 98% on study day 2 demonstrating that the combination had an excellent immediate curative effect. Efficacy was still > 90% for both AM and GM on days 9, 16, 23, 30 and 37 showing thus that the product had residual tick efficacy for a further 35 days.

### Tolerance

No clinical abnormalities were detected during the study. Only classic transitory cosmetic effects of a spot-on application like greasy appearance and/or clumping and/or spiking of the coat were observed during 24 h at both sites of administration.

## Discussion

In this study, a single treatment with the combination achieved more than 98% therapeutic efficacy for treating pre-existing infestation by *I. ricinus*. It was followed by a five weeks post-treatment residual efficacy as demonstrated by the prophylactic efficacy.

### Acaricidal effect

The acaricidal efficacy observed in this study against *I. ricinus* is similar to what is usually observed in equivalent experimental studies with recently approved acaricidal products and for which percentages of efficacy are available in the literature [14-18]. As regards the percentages of efficacy reported in the literature for fipronil-only and permethrin-only mono-products, the comparison of the results is in favor of the combination. Indeed, in a study comparing two topical formulations of fipronil registered within the European community at the same doses as the combination, the therapeutic efficacies of fipronil against *I. ricinus* varied between 93.8% and 98.8% whereas the prophylactic efficacies varied between 77.2% and 100.0% during the five weeks protection period [17].

In the same way, Endris *et al.* reported permethrin percentages of efficacy against *I. ricinus* ranging from 74.1 to 99.1% in a first study and from 90.3 to 99.5% in a second study [19,20]. In addition, the results published by Epe *et al.* for a spot-on combination of Imidacloprid 10% (w/v)/Permethrin 50% (w/v) - with respectively mainly insecticidal properties for imidacloprid and insecticidal and acaricidal properties for permethrin- are also in favor of the combination of two molecules with both acaricidal properties (fipronil/permethrin and fipronil/amitraz). In particular, the therapeutic efficacies of these fixed combination were better compared to reported therapeutic efficacy of the imidacloprid/permethrin combination (respectively 67% for the imidacloprid and permethrin combination *versus* 98% for the fipronil and permethrin combination as shown above and 97.9% for the fipronil and amitraz combination [18,21]).

The results published in the literature for fipronil and permethrin-based products are summarized in Table 5.

**Table 5:** **Published percentages of efficacy results of fipronil or permethrin based products against** ***Ixodes ricinus*** **(geometric means)** [17-21]

**Active ingredient(s)**	**Days after treatment**					
	**Day 2**	**Day 9**	**Day 16**	**Day 23**	**Day 30**	**Day 37**
**Fipronil + permethrin** ^**(a)**^	98%	100%	100%	100%	97%	96%
**Fipronil** ^**(b)**^	98.8%	100.0%	100.0%	100.0%	86.3%	77.2%
**Fipronil** ^**(c)**^	93.8%	100.0%	100.0%	98.9%	97.9%	94.1%
**Imidacloprid + permethrin** ^**(d)**^	67.0%	100.0%	100.0%	99.5%	98.7%	91.6%
**Fipronil + amitraz** ^**(e)**^	97.9%	100%	100%	98.1%	97.7%	96.3%
	**Day 2**	**Day 7**	**Day 14**	**Day 21**	**Day 28**	**Day 35**
**Permethrin** ^**(f)**^	99.1%	99.1%	95.9%	88.5%	87.1%	74.1%
**Permethrin** ^**(g)**^	-	96.3%	99.5%	90.7%	90.3%	91.3%

(a): Effitix®.

(b): Frontline ® Spot-on Dog Merial [17].

(c): Effipro® Spot-on Virbac SA [17].

(d): Imidacloprid 10% (w/v) / Permethrin 50% (w/v) Spot-on [18].

(e): Certifect^TM^ Merial Limited [21].

(f): Defend® Exspot® Treatment for dogs, Schering-Plough Animal Health Corp., 65% permethrin[19].

(g): Defend® Exspot® Treatment for dogs, Schering-Plough Animal Health Corp., 65% permethrin [20].

The combination of fipronil and permethrin shows globally numerically better therapeutic and prophylactic efficacies against *I. ricinus* than fipronil and permethrin mono- products. Consequently, combining fipronil and permethrin in the same formulation did not exhibit any antagonist effect; on the contrary, an improvement of the acaricidal efficacy against *I. ricinus* was shown.

### Repellent (anti-feeding) effect

In addition to its acaricidal properties, permethrin has been shown to have a strong repellent effect for a variety of arthropods including *I. ricinus* when applied to cloth or directly on the dog. Permethrin has been shown to be a contact repellent meaning that ectoparasites must come in contact with the molecule to be affected [22]. Miller *et al.* have shown that less than two hours exposure was sufficient to cause the ticks (*I. ricinus*) to move away from a permethrin-coated surface. Treatment of dogs with a 65% permethrin spot-on reduced tick numbers on dogs by 99.1% at 2 days, 99.0% at one week, 95.9% at two weeks, 88.5% at three weeks, 87.1% at four weeks post-treatment. In contrast, treatment of dogs with 9.7% fipronil did not reduce significantly tick numbers, after two hours exposure, as compared to the control group. These data indicate that *I. ricinus* are highly susceptible to permethrin. Permethrin provides a high level of protection and prevention in dogs by killing rapidly and repelling ticks before they can attach and begin feeding in particular when using highly concentrated formulations.

The design followed in this study evaluated only the acaricidal effect of the combination as 48 h time interval was allowed between the tick infestation and the evaluation of the efficacy. However the combination of permethrin and fipronil should show at least the same repellent properties as permethrin alone.

Consequently, with the use of a fipronil and permethrin combination, the protection against *I. ricinus* will combine an excellent killing effect together with repellent properties.

The primary concern about ticks is their ability to transmit vector-borne diseases. *B. burgdorferi,* the causal agent of Lyme disease is predominately transmitted by *Ixodes* ticks. The maximum transmission of the spirochete occurs between 48 and 72 h after the nymph attachment [17,23-25]. Similarly, the transmission of the human granulocytic ehrlichiosis agent, *A. phagocytophilum* was estimated to require at least 30 h in an experimental mice model infested with *Ixodes* nymphs [26]. Therefore, an excellent killing effect of *I. ricinus* together with a repellent effect are prudent measures to be included in an adequate protection strategy against Lyme disease transmission agents and potentially other vector-borne diseases.

### Resistance

Another speculative advantage of combining fipronil and permethrin may be to slow the development of resistance against both actives. If some parasites are less susceptible to one of the active ingredients, the second one will be active on them, and therefore prevent the emergence of these resistant-strains. This is particularly true when the mechanisms of resistance are independent and initially rare [27-29].

So far, no evidence of established resistance to fipronil and permethrin in dogs’ ticks and fleas has been reported in Europe [30].

If it can be proven by further studies that the administration of the combination could actually delay the development of resistance against fipronil and/or permethrin in Europe, this would be a huge step forward.

## Conclusions

The artificial infestation model with *I. ricinus* used for this study provided a challenge to assess the Effitix® acaricidal effect compared to natural conditions. This study has shown that Effitix® is a safe and an effective combination to treat and protect dogs from *I. ricinus* up to 37 days after administration. The excellent immediate efficacy of 98% evaluated 48 hours post Effitix treatment provides a rapid control of tick infestation. This combination can be used as a part of the strategy to control tick infestations, in particular when there is a risk of transmission of vector-borne diseases by ticks.
